# Changing treatment of hip fractures in Finland

**DOI:** 10.1007/s00402-024-05462-8

**Published:** 2024-08-28

**Authors:** Oskari K. Leino, Nora Forsbacka, Inari E. Laaksonen, Keijo T. Mäkelä, Markus Matilainen, Elina M. Ekman

**Affiliations:** 1 Department of Orthopaedics and Traumatology, Turku University Hospital, University of Turku, Turku, Finland; 2 Department of Biostatistics, Faculty of Medicine, University of Turku, Turku, Finland

**Keywords:** Hip fracture, Incidence, Treatment, Osteosynthesis, Arthroplasty

## Abstract

**Introduction:**

Hip fracture treatment should be as standardized and effective as possible, with emphasis on fast recovery and avoidance of complications, especially those leading to reoperations. There is accumulating evidence regarding the optimal treatment of hip fractures but reports of whether this has influenced treatment in the clinical setting are sparse. The objective of this study was to determine the trends of hip fracture incidence and treatment in Finland, with special regard to how we treat older patients compared to younger ones.

**Materials and methods:**

All operatively treated hip fractures in Finland between 1997 and 2018 were identified from a national administrative register. The incidence of these fractures and operations performed to treat them were calculated based on the adult population size.

**Results:**

Apart from a decline in the elderly age groups during the first half of the study period, the incidence of hip fractures remained relatively constant. However, the incidences of different operations changed significantly. In treatment of femoral neck fractures from 1997 to 2018, the incidence of cemented hemiarthroplasty (HA) increased from 41.1 to 59.9 per 100,000 person-years (10^*5*^) and hybrid total hip arthroplasty (THA) from 0.56 to 5.93 per 10^*5*^, while the incidence of internal fixation (IF) decreased, for instance screw fixation from 12.5 to 2.7 per 10^*5*^. The incidence of cementless HA decreased from 13.3 to 1.2 per 10^*5*^. These changes were much more pronounced in the elderly population and there was a statistically significant difference in the proportion of patients aged > 59 treated with cemented HA and IF in 1997 compared to 2018. For trochanteric and subtrochanteric fractures, treatment with intramedullary nails replaced extramedullary devices as the most common treatment method.

**Conclusions:**

The changes in treatment methods in Finland correspond to the increasing knowledge available about the optimal treatment of hip fractures and global treatment trends.

**Supplementary Information:**

The online version contains supplementary material available at 10.1007/s00402-024-05462-8.

## Introduction

Hip fractures are associated with significant mortality, morbidity, and functional impairment [1–3]. The incidence of hip fractures is decreasing in the Nordic countries and in the USA [4–6]. There is accumulating evidence regarding the optimal treatment of different hip fracture types. In general, hip fractures are treated operatively because the use of nonoperative treatment has been shown to lead to poor clinical results, even in non-displaced femoral neck fractures [7]. Operative treatment options for femoral neck fractures include IF and arthroplasty. Fractures of the trochanteric and subtrochanteric region are generally treated with IF, and extramedullary devices such as the Dynamic Hip Screw (DHS) have been challenged by intramedullary (IM) nailing, which has gained popularity in recent years [8]. This study aimed to determine the incidences of all operatively treated hip fractures in Finland by fracture type and treatment method between 1997 and 2018, with focus on the treatment of elderly patients compared to younger ones. To our knowledge, no other studies have reported national distributions of fracture types or treatment methods not only focusing on a specific age, population group or fracture type. Our expectation was that in femoral neck fractures, the use of cemented arthroplasty has increased and the use of IF has decreased in the elderly and that the use of IM nailing has become the most common treatment in trochanteric fractures.

## Materials and methods

**Fig. 1:**
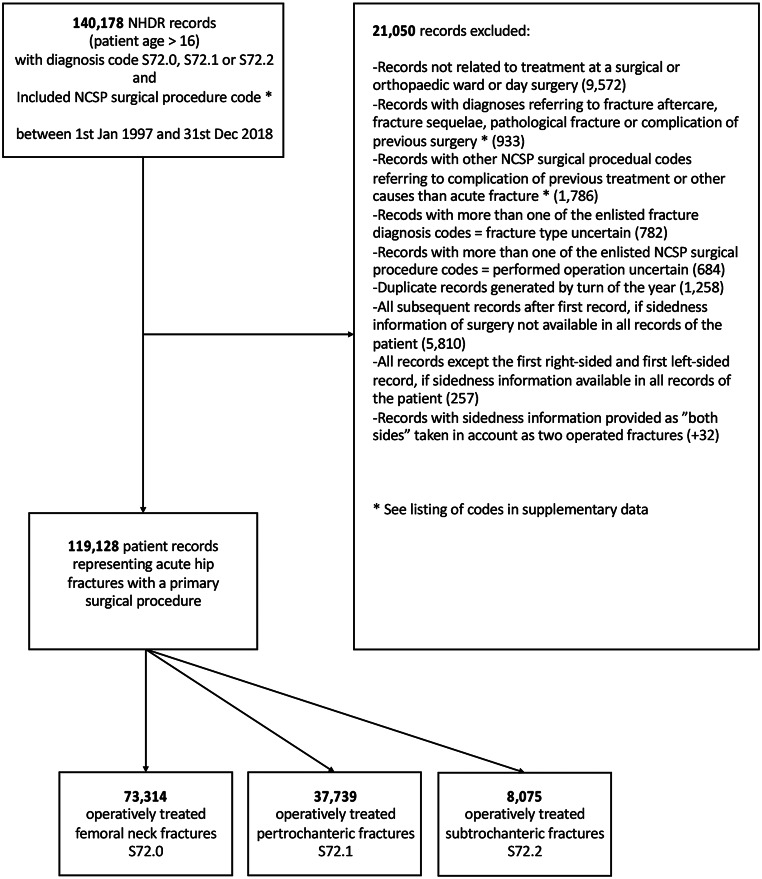
Exclusion flow chart (NHDR = National Hospital Discharge Register, NCSP = Nordic Classification of Surgical Procedures)

This study was based on data from the Finnish National Hospital Discharge Register (NHDR), which is maintained by the Finnish National Institute for Health and Welfare (THL). The register covers all patient records from private and public hospitals in Finland and has been in use since the 1960s. From each record, the register collects data on age, sex, domicile, external cause and type of injury, primary and secondary diagnoses, type of hospital (public or private), type of visit (emergency or inpatient care), specialty of service (internal medicine or orthopedics, etc.), and possible operations performed during the visit. The validity of the NHDR has been found to be good, especially in orthopedic trauma [9, 10]. The database was searched for all records with diagnosis codes S72.0 (Femoral neck fracture), S72.1 (Pertrochanteric femoral fracture), and S72.2 (Subtrochanteric femoral fracture) (International Classification of Diseases, ICD-10) from January 1, 1997 to December 31, 2018 because ICD-10 coding was introduced in 1996 in Finland. Only skeletally mature patients aged 16 and older were included. Exclusion of records was performed to identify and include only records with a primary surgical operation due to an acute hip fracture. (See exclusion flow chart [Fig. 1] and table of included Nordic Classification of Surgical Procedures [NCSP] procedural codes and excluded diagnosis and NCSP codes in the supplementary data.) The overall incidence and age- and sex-adjusted incidences were calculated based on the annual adult population size obtained from Statistics Finland [11]. Because the analysis was based on the entire population rather than cohort-based estimates, there was no need for 95% confidence interval calculations [4]. The significance of the trends in all incidences were assessed using the Mann-Kendall test (using R-software package Trend, version 1.1.6.). The Chi-squared test was used to assess the difference in proportions of cemented HA and IF for surgical neck fractures in the two oldest age groups, and the differences in incidences between age groups and sexes. P-values 0.05 or lower were considered statistically significant.

## Results

**Fig. 2:**
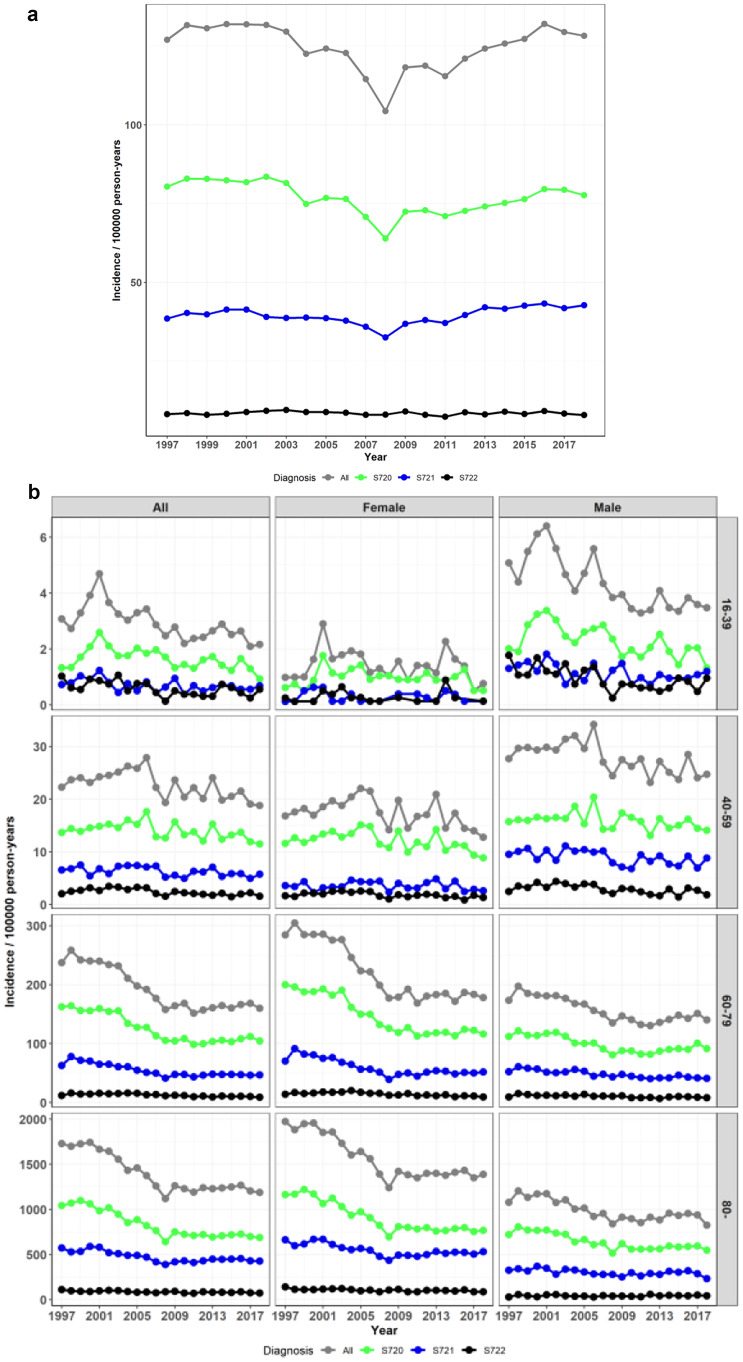
**a** Incidence of hip fractures by diagnosis **b** Incidence of hip fractures by diagnosis, sex, and age group (S720 = femoral neck fracture, S721 = pertrochanteric fracture S722 = subtrochanteric fracture)

We identified 119,128 patient records representing acute hip fractures with a primary surgical procedure. There were 73,314 operatively treated femoral neck fractures (61.5%), 37,739 operatively treated pertrochanteric fractures (31.7%), and 8,075 operatively treated subtrochanteric fractures (6.8%). During 1997–2018, no significant change was detected in the overall incidences of these fractures (Mann-Kendall test p-value 0.5 meaning no significant trend). The 1997 and 2018 incidences per 100,000 person-years (10^*5*^) of all operatively treated hip fractures were 127.0 vs. 128.3 per 10^*5*^, 80.4 vs. 77.7 per 10^*5*^ for femoral neck fractures, 38.5 vs. 42.8 per 10^*5*^ for pertrochanteric fractures, and 8.13 vs. 7.85 per 10^*5*^ for subtrochanteric fractures, respectively. (Fig. 2a).

The incidence was higher in the elderly age groups and among women in the elderly age groups (all p-values from tests between all the age groups and between the sexes in the two oldest age groups < 0.001). For instance, while the unadjusted incidence of all operatively treated hip fractures in 2018 was 128.3 per 10^*5*^, the 2018 incidence in patients aged 16–39 was 2.2 per 10^*5*^ (women 0.8 / men 3.5), 18.8 per 10^*5*^ in 40–59-year-old patients (w 12.8 / m 24.7), 160.1 per 10^*5*^ in 60–79-year-old patients (w 177.8 / m 140.3), and 1,189.4 per 10^*5*^ in > 79-year-old patients (w 1,389.5 / m 824.7). The incidence of all operatively treated hip fractures declined in the two oldest age groups during the first half of the study period. In 1997, the incidence in patients aged 60–79 and > 79 years were 237.0 per 10^*5*^ (w 284.2 / m 173.5) and 1,728.3 per 10^*5*^ (w 1,971.7 / m 1,079.4), respectively. (Fig. 2b). The trend was significant from 1997 to 2008 (*p* < 0.001 in both age groups), but not from 2008 to 2018 (*p* = 0.4 and *p* = 0.8, respectively).

### Treatment of femoral neck fractures

**Fig. 3:**
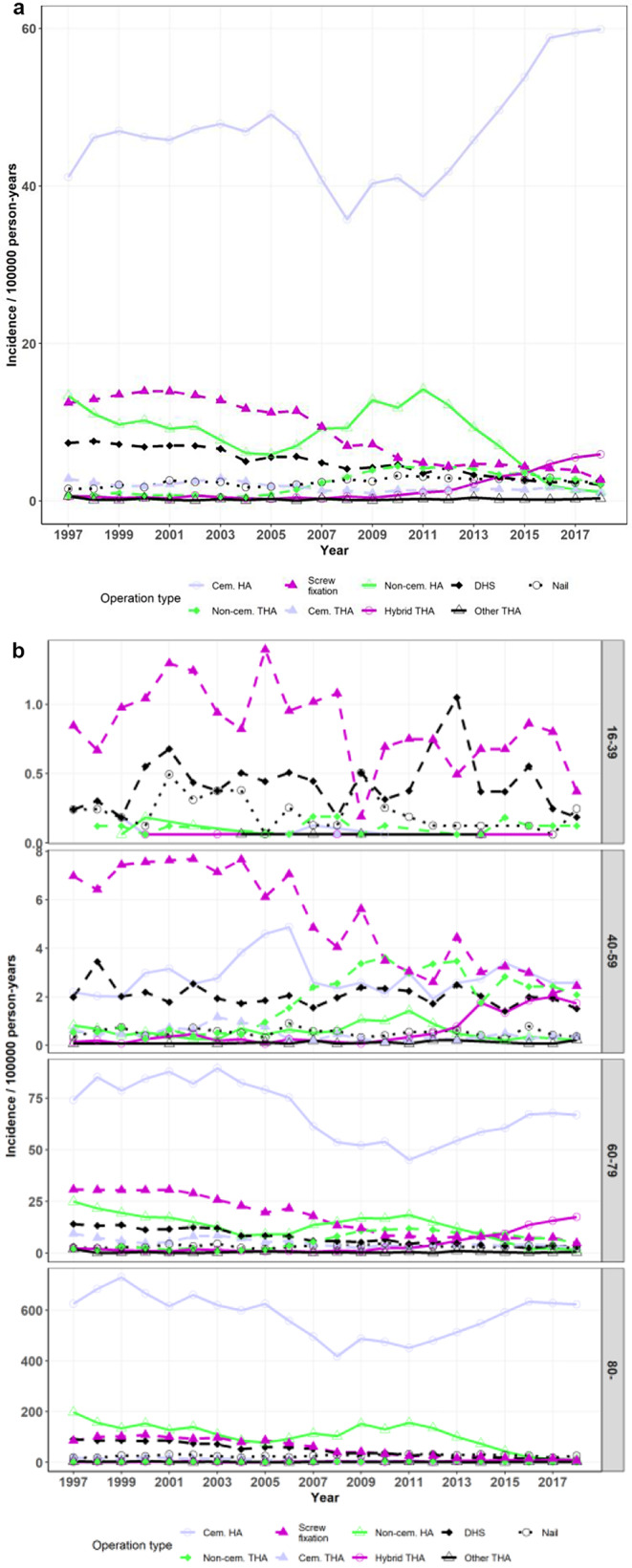
**a** Incidence of operations performed for femoral neck fractures by operation type **b** Incidence of operations performed for femoral neck fractures by operation type and age group (HA = Hemiarthroplasty, DHS = Dynamic Hip Screw, THA = Total Hip Arthroplasty, Cem. = Cemented)

A total of 44,813 cemented HAs, 7,946 cementless HAs, 1,504 hybrid THAs, 2,154 cementless THAs, 8,118 screw fixations, and 4,595 DHS fixations were performed to treat femoral neck fractures. In 1997–2018, the incidence of cemented HA increased from 41.1 to 59.9 per 10^*5*^ (*p* = 0.091) and hybrid THA increased from 0.56 to 5.93 per 10^*5*^ (*p* < 0.001), while cementless HA decreased from 13.3 to 1.2 per 10^*5*^ (*p* = 0.018). The incidence of cementless THA increased from 0.66 per 10^*5*^ in 1997, peaking at 4.40 per 10^*5*^ in 2010 (1997 to 2010 trend *p* = 0.003), and then decreased to 2.12 per 10^*5*^ in 2018 (2010 to 2018 trend *p* = 0.001). The use of screw fixation decreased from 12.5 to 2.7 per 10^*5*^ (*p* < 0.001) and the use of DHS fixation decreased from 7.35 to 2.06 per 10^*5*^ (*p* < 0.001) (Fig. 3a and b). Regarding the differences in treatment methods between age groups see Table 1. The shift toward cemented stems was clear in patients aged > 59, whereas the treatment methods remained more heterogeneous in younger patients. In age group 16–39, the incidences of all other arthroplasties were mostly 0 with a few sporadic years of incidence up to 0.18 per 10^*5*^ (mean incidence for all 0.01) while cementless THA incidence was more constant varying between 0 and 0.19 per 10^*5*^ (mean 0.08). Despite the fact that the change in incidence of cemented HA did not quite reach statistical significance in the general population, in the age group 60–79, there was a statistically significant difference in the proportion of patients treated with cemented HA in 1997 (46%) compared to 2018 (64%) (*p* < 0.001). In the same age group, there was a corresponding statistically significant difference in the proportion of IF patients in 1997 (29%) compared to 2018 (10%) (*p* < 0.001). The same was true for the age group > 79 (cemented HA 1997: 60% vs. 2018: 90% (*p* < 0.001), and IF 1997: 19% vs. 2018: 6% (*p* < 0.001)).

**Table 1:**
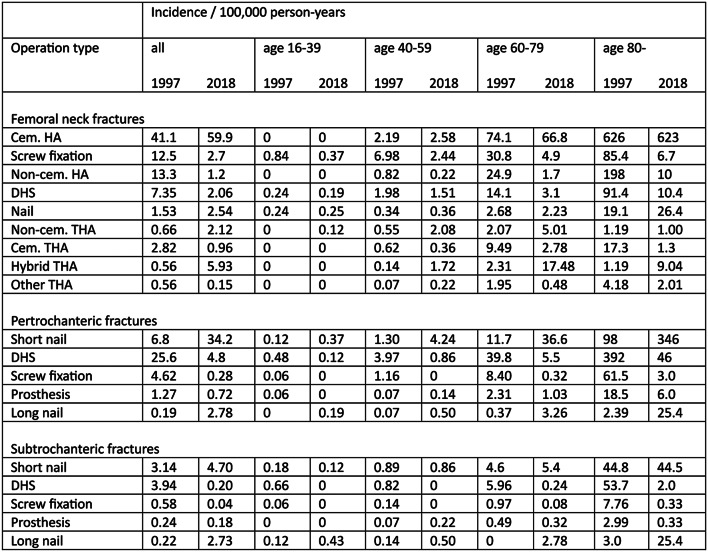
1997 and 2018 incidences of operation types by age group (HA = hemiarthroplasty, DHS = dynamic hip screw, THA = total hip arthroplasty, Cem. = cemented)

### Treatment of pertrochanteric fractures

**Fig. 4:**
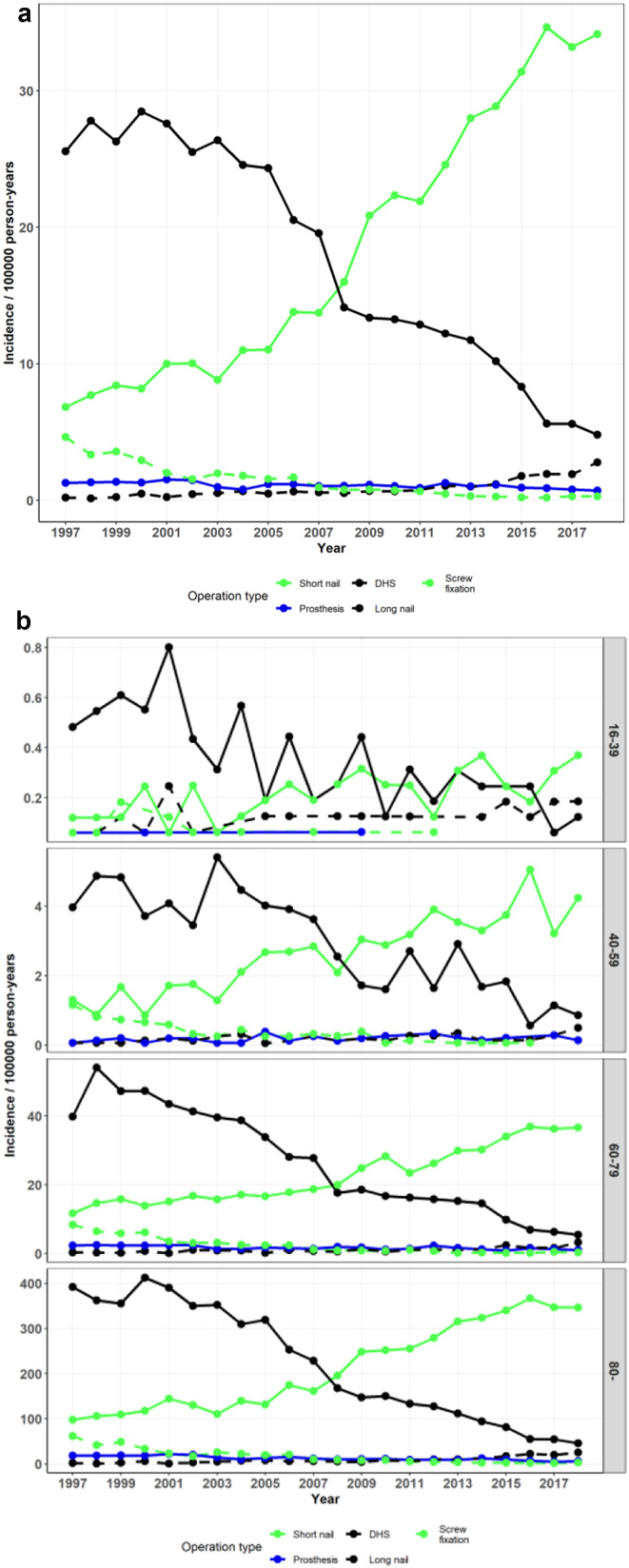
**a** Incidence of operations performed for pertrochanteric fractures by operation type **b** Incidence of operations performed for pertrochanteric fractures by operation type and age group (DHS = Dynamic Hip Screw)

Records indicated that 17,917 short IM nailings, 16,625 DHS fixations, 1,308 screw fixations, 1,050 arthroplasties, and 839 long IM nailings were performed to treat pertrochanteric fractures. The incidence of DHS use for pertrochanteric fractures decreased in 1997–2018 from 25.6 to 4.8 per 10^*5*^ (*p* < 0.001) and that of short IM nails increased from 6.8 to 34.2 per 10^*5*^ (*p* < 0.001). (Figure 4a and b; Table 1).

### Treatment of subtrochanteric fractures

**Fig. 5:**
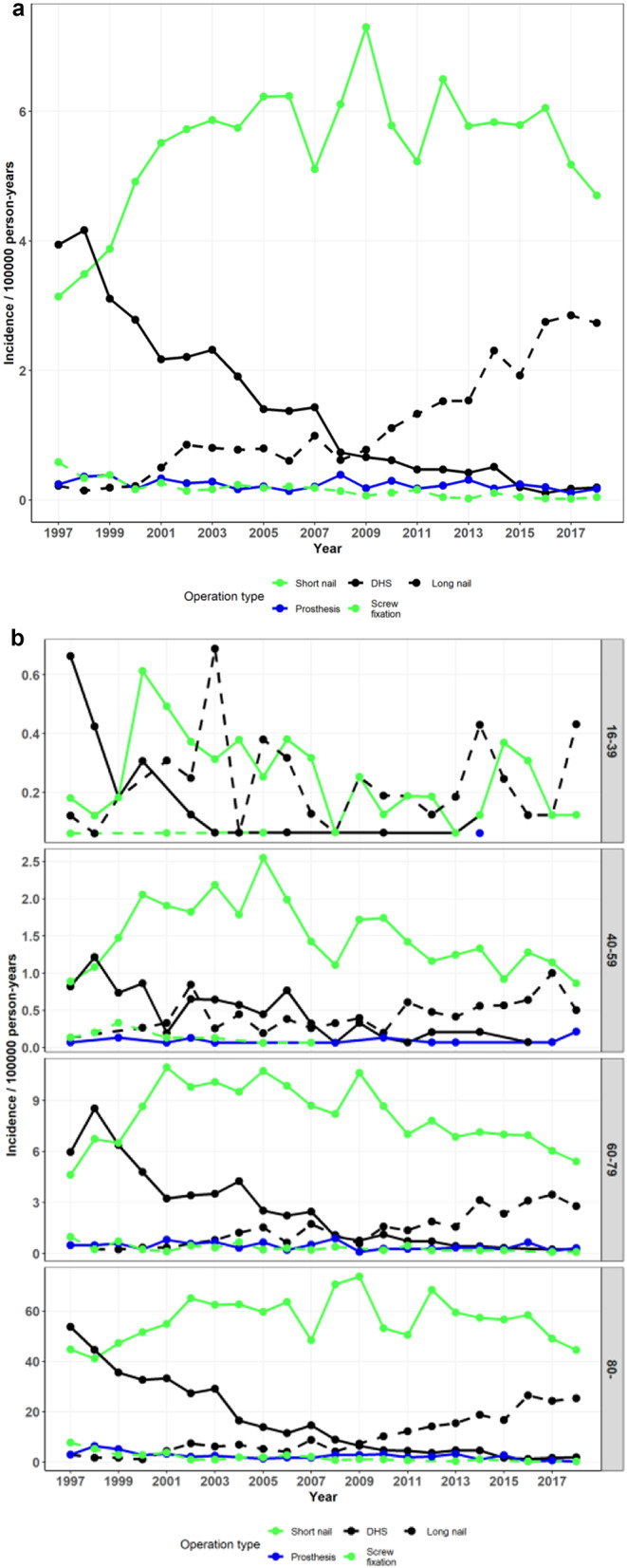
**a** Incidence of operations performed for subtrochanteric fractures by operation type **b** Incidence of operations performed for pertrochanteric fractures by operation type and age group (DHS = Dynamic Hip Screw)

A total of 5,231 short IM nailings, 1,326 DHS fixations, 1,136 long IM nailings, and 227 arthroplasties were performed to treat subtrochanteric fractures. The incidence of these fractures treated with short IM nails increased in 1997–2018 from 3.14 to 4.70 per 10^*5*^ (*p* = 0.063) and those treated with DHS decreased from 3.94 to 0.20 per 10^*5*^ (*p* < 0.001). The incidence of long IM nails increased toward the end of the study period from 0.22 to 2.73 per 10^*5*^ (*p* < 0.001) and the most pronounced increase was in age group > 79. (Figure 5a and b; Table 1).

The use of arthroplasty for pertrochanteric and subtrochanteric fractures remained marginal (trend *p* = 0.063 and *p* < 0.001, respectively) throughout the period (numbering 1,050 for pertrochanteric and 227 for subtrochanteric fractures) with respective incidences ranging between 0.72 and 1.53 per 10^*5*^ (mean 1.10) and 0.11–0.39 per 10^*5*^ (mean 0.24) (Figs. 4a and b and 5a and b; Table 1).

## Discussion

We found that apart from a significant decline in the elderly age groups during the first half of the study period, the incidence of hip fractures remained relatively constant. Despite this, most treatment methods changed significantly. For femoral neck fractures, we see that cemented HA became more popular while use of IF and cementless HA decreased significantly, especially in the older age groups. Although THA numbers were small compared to HA, the increase in hybrid THA incidence was almost 1,000% during the period. For pertrochanteric and subtrochanteric fractures, the use of IM nails effectively replaced DHS or comparable extramedullary devices.

The fractures in this study most often affected elderly women, whereas younger patients were predominantly men, mirroring previous epidemiological studies [4–6]. The decrease in operatively treated hip fracture incidence in the two oldest age groups during the first half of the study period corresponds to previously reported age-adjusted incidences of hip fractures from Finland by Kannus et al. [4] and other epidemiological studies reporting decreasing hip fracture incidence especially among the elderly female population [5, 6]. It seems that this decline has hence evened out. One should also note that hip fracture numbers are increasing despite the decreasing incidence rates due to the growing elderly populations in developed countries.

### Treatment of femoral neck fractures

Due to the risk of avascular necrosis of the femoral head in displaced femoral neck fractures, IF is generally recommended primarily for non-displaced and minimally displaced fractures [12]. IF has been compared with arthroplasty, which has been shown to lead to more predictable outcomes and less reoperations, even in cases of non-displaced fractures in the elderly [7, 13–15]. A recent Swedish study reported that 1 in 10 patients aged > 59 treated with IF for non-displaced fractures had revision to arthroplasty within five years [16]. Others report reoperation rates of 18% [17] to as high as 27% [13] after IF. A 2016 review concluded that arthroplasty should be the treatment of choice for elderly patients [18]. This notion is further reinforced by a recent Cochrane review [19]. Our results show that during 1997–2018, the use of IF significantly decreased in Finnish patients, especially those aged > 59. Similar results have been reported from Denmark in 1997–2017, where the use of IF decreased from 48 to 25% of all operatively treated femoral neck fractures in patients aged > 59 [5]. In Finland, the use of screw fixation decreased even in younger patients. This trend may in part be explained by relative increase in arthroplasty and DHS use in this age group. Another possible explanation is the introduction of novel implants such as the Femoral Neck System (FNS) by Synthes^®^, an evolution of the sliding hip screw design of the DHS, which is offered as an alternative for DHS or screw fixation. The NCSP system does not currently differentiate between the DHS and FNS, but because the FNS was introduced in 2018, there were few, if any, fractures treated with FNS included in the study.

There is accumulating evidence promoting the use of cemented stems for elderly patients both in HA and THA. The benefits of cemented stems in hip arthroplasty include less risk of periprosthetic fractures and fewer infection complications based on large register datasets [20–22]. These risk differences may be even more pronounced in fracture patients, who are typically elderly and more co-morbid compared to elective THA patients. Cementless HA has been shown to be at higher risk of revision than cemented HA [23], even when infection has been ruled out as cause [24]. A recent British multicenter study comparing cemented with cementless HA even demonstrated significantly better quality of life alongside decreased risk of periprosthetic fracture in patients treated with cemented HA [25]. Previous Finnish reports of increasing use of cementless stems in older fracture patients [26] were in conflict with available evidence regarding the subject. According to our data, this increase was short-lived as the use of cementless HA has significantly decreased. The use of hybrid THA gained popularity especially in patients aged 60–79, but even in younger patients aged 40–59. These younger fracture patients differ from elective THA patients as alcoholism and other conditions associated with higher risks of hip fracture and surgical complications are common, thereby influencing the choice of treatment method. In this age group, the first-hand choice for elective THA patients with good bone quality is a cementless stem [27].

When comparing THA with HA in fracture surgery, better functional outcome and lower revision rates in favor of THA have been reported despite a slightly higher risk of dislocation [28]. Dislocation risk reduction can be achieved by avoiding a posterior approach and using bipolar components in selected cases [29, 30]. It has been suggested that THA should be preferred in < 80-year-olds with more than four years life expectancy [31] and considered for patients who are ambulatory without assisting devices with more than two years expected independence in activities of daily living [32]. Others argue that there is no difference in clinical outcome when treating fitter elderly patients [33] and that the possible differences are clinically nonsignificant [34, 35]. Regardless of these, there are reports of increasing use of THA for hip fractures in the US [36] and in European countries such as Norway [13]. According to Miller et al., the use of THA among all operations performed for femoral neck fractures in patients < 65 increased in the US from 1.4 to 13.1% during 1999–2011 [36]. We see a corresponding trend of increasing THA use in patients aged 40–59, where based on the current data cementless and hybrid THA use increased from 5 to 33% of all operations performed for femoral neck fractures during 1997–2018. In Finland, the use of cemented THA has become increasingly rare thanks to the advances in acetabular polyethylene liner technology as indicated by the Finnish Arthroplasty Register (FAR) (FAR: Total Hip Arthroplasty: Annual counts, http://www.thl.fi/far/).

### Treatment of pertrochanteric and subtrochanteric fractures.

The use of DHS decreased markedly in Finland while that of short IM nails increased. Both trends were statistically significant. The figures crossed in 2008–2009 and the trend continued thereafter. The trend was similar but less apparent in the youngest patients, which may be due to the far lower incidence in this population (the absolute numbers of operations were small in age group 16–39, with 124 DHSs and 76 short IM nails during the entire period) and perhaps the preference of avoiding the trochanter-tip mutilating entry of IM nails. A trend of favoring IM nails has also been observed in the US, where in 1999–2006, the IM nailing rate of intertrochanteric fractures increased from 3 to 67% [8]. In Denmark, the choice of implant in trochanteric fractures also shifted toward IM nailing in 1997–2017 [5]. There are reports of less implant failures and reoperations favoring IM nailing to extramedullary fixation, although some unresolved issues regarding implant type superiority and cost effectiveness remain [37, 38].

Our finding that subtrochanteric femoral fractures were largely treated with short IM nails is interesting. This may be partially explained by the inter-observer variation of fracture classification as per- and intertrochanteric or subtrochanteric, a subject we sometimes find ourselves debating in radiograph meetings. Some of these subtrochanteric fractures are most likely in fact pertrochanteric. The AO/OTA classification of femoral fractures separates fractures of the trochanteric area into stable and unstable subtypes, guiding the decision between implant choices. However, the inter-observer reliability of this classification and other fracture classification systems of the trochanteric area has been questioned [39]. Another explanation might be the use of medium-length nails, such as the 240–280 mm-long Proximal Femoral Nail Antirotation by Synthes^®^, which is indicated for “high subtrochanteric fractures” according to the manufacturer. The NSPC system divides IM nailing of the femur into two categories: NFJ54 (Intramedullary Nailing of Upper Femur) and NFJ60 (Intramedullary Nailing of Femur). These procedural codes are quite ambiguous and might further explain our findings.

There are global reports describing the use and results of hip arthroplasty to treat pertrochanteric and subtrochanteric fractures [40, 41]. In Finland, the use of arthroplasty was marginal in these fractures but not non-existent, as was our expectation. Despite the trend test indicating a statistically significant change in cases of subtrochanteric fractures, this can in our view not be interpreted as a change in clinical paradigm due to the very small number of procedures. We suspect the incidence might be even smaller, as some periprosthetic fractures of the proximal femur may have been erroneously coded as proximal femur fractures, despite our efforts to screen out such cases based on additional diagnosis and procedural codes in the records.

### Limitations

This is an observational descriptive study based on an administrative register. The process of identifying acute hip fractures from the vast quantity of patient records was done by extensive exclusion as described in the Materials and Methods section. Records in which the exact fracture diagnosis code or surgical procedure code were not clearly ascertainable were excluded. Data on fracture classification other than ICD-10 codes or more specific patient-related issues were not available. This is important as we can only report what has been done, not the reasoning behind the chosen treatment method. The observed changes in treatment methods are under the presumption that fracture pattern distributions and other patient related characteristics have remained relatively stable during the period. We look forward to future studies from countries with high quality fracture registers such as Sweden, where national fracture specific data has been collected for over a decade now, as an answer to this issue [42]. We found that hip fracture diagnosis codes often lingered in subsequent patient records, which is probably a result of the comorbidity and disability that follow this devastating injury, especially in the elderly. Accordingly, we decided to include only the first recorded operatively treated injury of every patient or the first right- and first left-sided operatively treated injury when sidedness data were available. Moreover, identifying nonoperatively treated hip fractures, which was something we were originally hoping to report, was clearly going to be impossible. We know that a small portion of hip fractures are treated nonoperatively, such as in some cases of trochanter tip avulsions and cases where the patient is in terminal care. However, as the portion of these nonoperatively treated hip fractures is small, we find that the trends in operatively treated hip fracture incidence quite reliably represent the trends in hip fracture incidence in general. This also seems to be the case when comparing our results with previously published data on Finnish hip fracture incidence [4]. A clear strength of this study as well as other national register studies is its power in size as it includes the entire population of a country. The results are generalizable to other developed western countries. Finally, we wish to emphasize that our aim was only to report trends in the incidence of operatively treated hip fractures over time, not to report or compare clinical results or give treatment recommendations.

## Conclusion

In Finland, femoral neck fractures were increasingly treated with arthroplasty with cemented stems and the use of osteosynthesis decreased. Trochanteric and subtrochanteric fractures were increasingly treated with IM nailing while extramedullary devices became less popular. These findings are in line with current scientific evidence concerning the optimal treatment of femoral neck fractures and although the optimal treatment of trochanteric fractures remains controversial, Finnish practices correspond to global treatment trends.

## Electronic supplementary material

Below is the link to the electronic supplementary material.

Supplementary Material 1
