# Supplementary material for: Changing treatment of hip fractures in Finland
Source: Arch Orthop Trauma Surg. 2024 Aug 28;144(8):3469–78. doi: 10.1007/s00402-024-05462-8 (PMC11417085; doi:10.1007/s00402-024-05462-8)
Supplement: Supplementary file 1 — Supplementary Material 1 [file 402_2024_5462_MOESM1_ESM.docx]

**Included NCSP (Nordic Classification of Surgical Procedures) procedural codes**

NCSP procedural codes:

NFB10 Primary partial prosthetic replacement of hip joint not using cement

NFB20 Primary partial prosthetic replacement of hip joint using cement

NFB30 Primary total prosthetic replacement of hip joint not using cement

NFB40 Primary total prosthetic replacement of hip joint using hybrid technique

NFB50 Primary total prosthetic replacement of hip joint using cement

NFB60 Demanding prosthetic replacement of hip

NFB99 Other primary prosthetic replacement of hip joint

NFJ50 Internal fixation of fracture of neck of femur with nail or screw

NFJ52 Internal fixation of fracture of upper femur with screws and sideplate

NFJ54 Internal fixation of fracture of upper femur with intramedullary nail

NFJ60 Internal fixation of fracture of other parts of femur with intramedullary nail

**ICD-10 Diagnosis and NCSP procedural codes leading to exclusion of record:**

ICD-10 Diagnosis codes:

M84.0 Malunion of fracture

M84.1 Nonunion of fracture [pseudarthrosis]

M84.2 Delayed union of fracture

M84.3 Stress fracture, not elsewhere classified

M84.4 Pathological fracture, not elsewhere classified

M90.7 Fracture of bone in neoplastic disease

M96.6 Fracture of bone following insertion of orthopaedic implant, joint prosthesis, or bone plate

T81.0 Haemorrhage and haematoma complicating a procedure, not elsewhere classified

T81.3 Disruption of operation wound, not elsewhere classified

T81.4 Infection following a procedure, not elsewhere classified

T81.58 Acute reaction to foreign substance accidentally left during a procedure

T81.7 Vascular complications following a procedure, not elsewhere classified

T81.8 Other complications of procedures, not elsewhere classified

T81.9 Unspecified complication of procedure

T84.0 Mechanical complication of internal joint prosthesis

T84.1 Mechanical complication of internal fixation device of bones of limb

T84.2 Mechanical complication of internal fixation device of other bones

T84.3 Mechanical complication of other bone devices, implants and grafts

T84.4 Mechanical complication of other internal orthopaedic devices, implants and grafts

T84.5 Infection and inflammatory reaction due to internal joint prosthesis

T84.68 Infection and inflammatory reaction due to internal fixation device [any site]

T84.7 Infection and inflammatory reaction due to other internal orthopaedic prosthetic devices, implants and grafts

T84.8 Other complications of internal orthopaedic prosthetic devices, implants and grafts

T84.9 Unspecified complication of internal orthopaedic prosthetic device, implant and graft

T93.1 Sequelae of fracture of femur

NCSP procedural codes:

NFU00 Removal of partial prosthesis from hip joint

NFU20 Removal of internal fixation device from femur

NFU99 Removal of other implant from hip joint or femur

NFC20 Secondary implantation of hip prosthesis

NFC30 Repair of hip prosthesis by bone transplant to femur

NFC40 Repair of hip prosthesis by bone transplant to acetabulum

NFC50 Repair of hip prothesis by bone transplant to femur and acetabulum

NFJ84 Refixation of fracture of femur

NFJ86 Late operation for fracture of femur to promote bone formation
